# BMP4 depletion by miR-200 inhibits tumorigenesis and metastasis of lung adenocarcinoma cells

**DOI:** 10.1186/s12943-015-0441-y

**Published:** 2015-09-22

**Authors:** Jeong Seon Kim, Jonathan M. Kurie, Young-Ho Ahn

**Affiliations:** Department of Molecular Medicine and Tissue Injury Defense Research Center, Ewha Womans University School of Medicine, Seoul, South Korea; Department of Thoracic/Head and Neck Medical Oncology, The University of Texas MD Anderson Cancer Center, Houston, TX USA

**Keywords:** BMP4, miR-200, Lung cancer, Metastasis

## Abstract

**Background:**

MicroRNA-200 (miR-200) suppresses the epithelial-mesenchymal transition of various cancer cells, including lung adenocarcinoma cells. We found that bone morphogenetic protein 4 (BMP4) was decreased in miR-200-overexpressing cells and epithelial-like lung cancer cells. In this study, we investigated the mechanism and role of BMP4 depletion by miR-200 in murine lung adenocarcinoma cells.

**Methods:**

BMP4 expression levels in murine lung cancer cells were measured by quantitative reverse transcription-PCR (qRT-PCR) and Western blotting. Promoter and 3′-untranslated region (UTR) luciferase reporter assays were performed to discover the mechanism of regulation of BMP4 by miR-200. Murine lung cancer cells were transfected with *Bmp4* shRNAs, which were then injected into syngeneic mice to measure their tumorigenic and metastatic potential and cultured on Matrigel to study the influence of BMP4 on 3-D acinus formation.

**Results:**

miR-200 down-regulated BMP4 via direct targeting of the GATA4 and GATA6 transcription factors that stimulate *Bmp4* transcription. BMP4 up-regulated JAG2, an upstream factor of miR-200; therefore, JAG2, miR-200, and BMP4 form a regulatory loop. *Bmp4* knockdown suppressed cancer cell growth, migration, and invasion and inhibited tumorigenesis and metastasis of lung cancer cells when injected into syngeneic mice. In addition, BMP4 was required for normal acinus formation in Matrigel 3-D culture of murine lung cancer cells, which may be mediated by MYH10, a downstream target of BMP4.

**Conclusion:**

BMP4 functions as a pro-tumorigenic factor in a murine lung cancer model, and its transcription is regulated by miR-200 and GATA4/6. Thus, we propose that BMP4 and its antagonists may be suitable therapeutic targets for the treatment of lung cancer.

**Electronic supplementary material:**

The online version of this article (doi:10.1186/s12943-015-0441-y) contains supplementary material, which is available to authorized users.

## Background

Lung cancer is the leading cause of cancer-related death in both men and women worldwide [1]. Although effective cancer therapies, including personalized medicine using epidermal growth factor receptor (EGFR) tyrosine kinase inhibitors and anaplastic lymphoma kinase (ALK) inhibitors, have significantly improved the prognosis and survival of lung cancer patients [2], the 5-year survival rate of patients with metastatic lung cancer is still less than 5 % [1]. Clearly, understanding the biological processes that underlie cancer metastasis will be crucial to achieving long-term survival for lung cancer patients.

During metastasis, cancer cells from a primary tumor are disseminated throughout the body through sequential steps: local invasion, intravasation, survival in circulation, extravasation into distant organs, and colonization at secondary sites [3]. Local invasion begins when a subgroup of tumor cells undergoes epithelial-mesenchymal transition (EMT), characterized by the loss of cell-cell attachments and apical-basal polarity and the appearance of mesenchymal differentiation properties [3]. To prevent metastasis and cancer recurrence in patients, it is essential to identify the mechanisms triggering the EMT process.

To study metastasis and EMT in lung cancer, we and other groups generated a mouse lung cancer model that develops spontaneous lung tumors with a high incidence of metastases due to combined mutations of *Kras* (*Kras*^G12D^) and *Trp53* (*Trp53*^R172HΔG^) [4, 5]. From the tumors of these KRAS/p53-mutant mice (KP mice), we also established several murine lung cancer cell lines (KP cells) that exhibit various levels of metastatic potential mainly regulated by ZEB1 and microRNA-200 (miR-200) [6]. ZEB1 and miR-200 form a double-negative feedback loop [7, 8] that plays a key role in determining the metastatic fate of epithelial cancers through the regulation of downstream target genes and microRNAs (miRNAs).

**Fig. 1:**
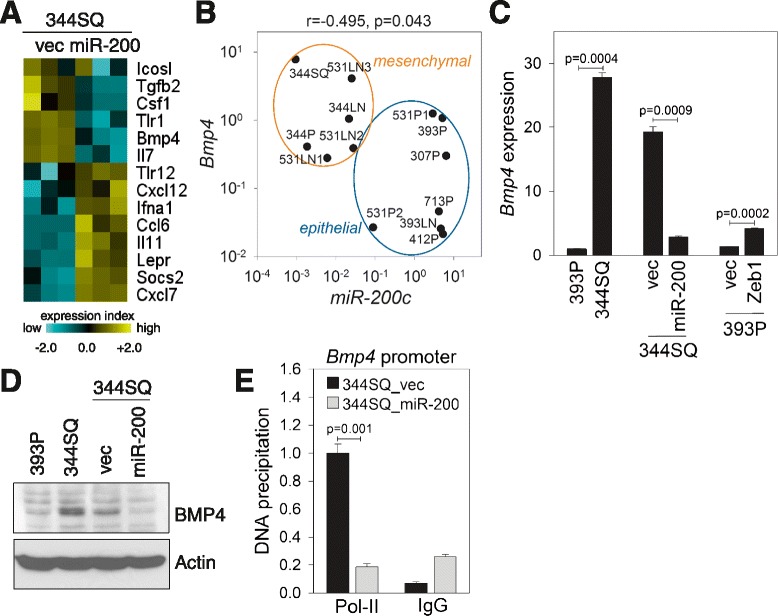
BMP4 is down-regulated in 344SQ_miR-200 cells. **a** The expression profile of immune-related genes in 344SQ_vector (vec) or 344SQ_miR-200 cells. Yellow: genes up-regulated by miR-200 overexpression; blue: genes down-regulated by miR-200 overexpression. **b** Quantitative RT-PCR (qRT-PCR) of *Bmp4* and miR-200c in 13 murine lung adenocarcinoma cells. Cells were grouped into epithelial- or mesenchymal-like cells on the basis of the expression of EMT markers [13]. r and p, one-tailed Spearman’s rank correlation test. **c** qRT-PCR of *Bmp4* in 393P, 344SQ, 344SQ_miR-200, 393P_Zeb1, and their control cells (vec). Expression levels were normalized to that of 393P (=1.0). Mean + SD, n = 3; p, two-tailed Student’s *t*-test. **d** BMP4 Western blot in 393P, 344SQ, 344SQ_vec, and 344SQ_miR-200 cells. Actin was used as a loading control. **e** RNA polymerase II (Pol-II) chromatin immunoprecipitation assay on the *Bmp4* promoter region in 344SQ_vec and 344SQ_miR-200 cells. Bars denote DNA precipitation (% of input) from each sample. IgG was used as a negative control. Mean + SD, n = 3; p, two-tailed Student’s *t*-test

To identify genes downstream of miR-200, we performed microarray-based transcriptional profiling in KP cells overexpressing a miR-200 cluster, miR-200b/200a/429 [6]. Ectopic expression of a miR-200 cluster (miR-200b/200a/429) in a highly metastatic murine lung adenocarcinoma cell line (344SQ) blocks EMT and metastasis and induces global gene expression changes [6]. Transcriptional profiling revealed that expression of several cytokines/chemokines and their receptors is up- or down-regulated by miR-200 overexpression (Fig. 1a). Among hundreds of genes down-regulated by miR-200, we focused on bone morphogenetic protein 4 (BMP4), a member of the transforming growth factor β (TGF-β) superfamily, which is involved not only in early embryonic development but also in cellular growth, differentiation, and tumorigenesis [9]. Interestingly, BMP4 plays opposing roles in tumorigenesis and metastasis depending on cellular context. For example, BMP4 causes tumor-initiating cell depletion and inhibits tumorigenesis in glioblastomas [10]; however, BMP4 induces EMT in pancreatic cancer cells [11].

In the present study, we showed that miR-200 suppresses BMP4 indirectly through the GATA4 and GATA6 transcription factors and that BMP4 knockdown inhibits cancer cell growth, migration, invasion, and metastasis. Based on these findings, we concluded that BMP4 is one of the key downstream factors that mediate miR-200’s effects on lung tumorigenesis and metastasis and is a candidate target for directed cancer therapy.

## Results

### Expression of BMP4 is inversely correlated with that of miR-200

To clarify the roles of BMP4 in lung tumorigenesis and metastasis, we analyzed the expression levels of *Bmp4* mRNA and miR-200 family members in 13 KP cell lines (Fig. 1b and Additional file 1: Figure S1). Mesenchymal-like cells exhibited higher *Bmp4* mRNA expression than did epithelial-like cells, and *Bmp4* tended to correlate negatively with miR-200 members. However, only the correlation between *Bmp4* and miR-200c was statistically significant (r = -0.495, *p* = 0.043; one-tailed Spearman’s rank correlation), which might be due to an insufficient sample size (n = 13). Similar negative correlations between *Bmp4* and miR-200 members were also observed in human lung adenocarcinomas and breast invasive carcinomas (Additional file 1: Figure S2 and S3). ZEB1, a transcription suppressor of miR-200, enhanced *Bmp4* expression in a low metastatic lung cancer cell line (393P), but miR-200 suppressed *Bmp4* expression in 344SQ (Fig. 1c) and 531LN2 (Additional file 1: Figure S4), which was confirmed by Western blotting (Fig. 1d). The effect of miR-200 on BMP4 expression may not be mediated via direct 3′ -untranslated region (UTR) binding because there is no putative miR-200 binding site on *Bmp4*’s 3′ -UTR (http://www.targetscan.org), and less chromosomal DNA of the *Bmp4* promoter region was immunoprecipitated by an RNA polymerase II (Pol-II) antibody from 344SQ_miR-200 cells than from 344SQ_vec control cells (Fig. 1e), which clearly suggests transcriptional regulation of *Bmp4* mRNA expression by miR-200 via indirect mechanisms.

### miR-200 down-regulates *Bmp4* through GATA4 and GATA6

**Fig. 2:**
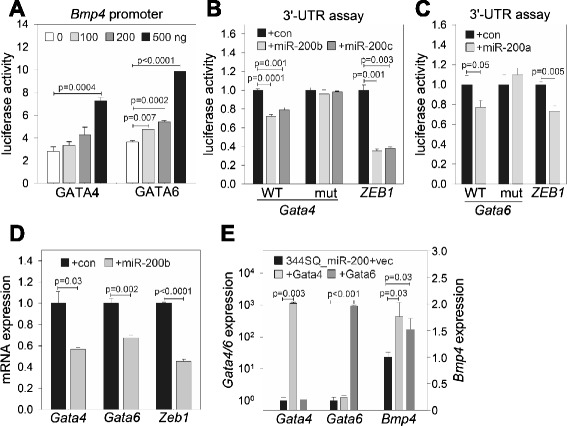
GATA4 and GATA6, transcription factors of BMP4, are miR-200 target genes. **a** Luciferase assay of *Bmp4* promoter activity. 393P cells were co-transfected with GATA4 or GATA6 expression vector and the *Bmp4* promoter reporter. Results were normalized by using a Renilla luciferase vector (pRL-TK). Mean + SD, n = 3; p, two-tailed Student’s *t*-test. **b**, **c** Luciferase assay of *Gata4* and *Gata6* 3′-UTR activity. 344SQ cells were co-transfected with control (con) or miR-200 mimics and 3′-UTR reporter constructs. Results were normalized by using a firefly luciferase vector (pGL3-con). *ZEB1* 3′-UTR construct was used as a positive control. Mean + SD, n = 3; p, two-tailed Student’s *t*-test. **d** qRT-PCR of *Gata4*, *Gata6*, and *Zeb1* in 344SQ cells transfected transiently with control (con) or miR-200b mimic. The expression levels were normalized to those of the control group (=1.0). Mean + SD, n = 3; p, two-tailed Student’s *t*-test. **e** *Bmp4* qRT-PCR in 344SQ_miR-200 cells transfected with GATA4 or GATA6. The expression levels were normalized to that of vector-transfected cells (=1.0). Mean + SD, n = 3; p, two-tailed Student’s *t*-test

To identify the mediators of miR-200’s suppressive effect on *Bmp4* expression, we first searched for putative transcriptional regulators that act on the *Bmp4* promoter. Previously, we reported that GATA factors are down-regulated by miR-200 [12, 13]. Moreover, GATA4 and GATA6 have been proven to be bona fide transcription factors for *Bmp4* [14], which was also confirmed in our system via luciferase reporter assays using a *Bmp4* promoter construct (Fig. 2a). Interestingly, *Gata4* and *Gata6* have conserved miR-200 binding sites on their 3′-UTRs: *Gata4* has a miR-200b/200c/429 site and *Gata6* has a miR-200a/141 site (http://www.targetscan.org). To test for direct interaction between miR-200 and these 3′-UTRs, we made *Gata4* and *Gata6* 3′ -UTR reporter constructs and performed luciferase assays after co-transfection with miR-200 mimics. miR-200b and 200c suppressed *Gata4* 3′ -UTR activity, and miR-200a inhibited *Gata6* 3′ -UTR activity as reported previously [13], which were restored by mutating the miR-200 binding sites on their 3′-UTRs (Fig. 2b and 2c). In addition, miR-200b repressed *Gata4* and *Gata6* mRNA expression in 344SQ cells (Fig. 2d), and ectopic GATA4 or GATA6 expression in 344SQ_miR-200 cells reinstated the *Bmp4* mRNA level suppressed by miR-200 (Fig. 2e). On the basis of these data, we propose that miR-200 down-regulates *Bmp4* through direct targeting of its transcription factors, GATA4 and GATA6.

### *Bmp4* knockdown suppresses migration, invasion, tumorigenesis, and metastasis of lung cancer cells

**Fig. 3:**
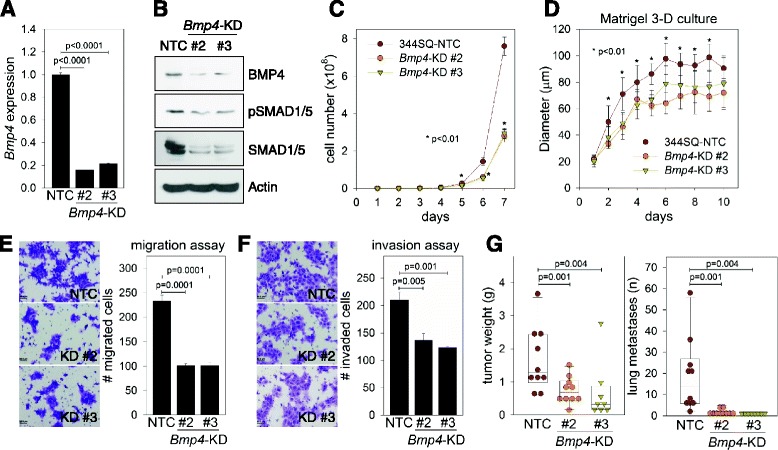
BMP4 knockdown suppresses cancer cell growth, migration, invasion, tumorigenesis, and metastasis. **a** qRT-PCR of *Bmp4* in 344SQ transfected with *Bmp4* shRNAs (KD #2 and #3) or nontargeting control (NTC) vector. The expression levels were normalized to that of NTC (=1.0). Mean + SD, n = 3; p, two-tailed Student’s *t*-test. **b** Western blot of BMP4, phospho-SMAD1/5 (pSMAD1/5), and total SMAD1/5 in 344SQ-*Bmp4*-KD cells. Actin was used as a loading control. (**C**) Cell numbers of 344SQ-*Bmp4*-KD and NTC cells over 7 days. Mean ± SD, n = 4; p, two-tailed Student’s *t*-test. (**D**) Diameters of 3-D acini of 344SQ-*Bmp4*-KD and NTC cells cultured on Matrigel for 10 days. Mean ± SD, n = 15; p, two-tailed Student’s *t*-test. **e**, **f** Migrated (**e**) and invaded (**f**) cells in Boyden chambers were photographed and counted. Mitomycin C (1 μg/mL) was added to the culture media to block cell proliferation. Mean + SD, n = 3; p, two-tailed Student’s *t*-test. **g** Primary tumor weight (*left*) and lung metastases (*right*) in syngeneic mice injected with 344SQ-*Bmp4*-KD and NTC cells. Box-and-whisker plots denote median and upper/lower quartiles + 1.5 × IQR (interquartile range). p, two-tailed Student’s *t*-test

Next, to investigate the biological role of *Bmp4* repression by miR-200, we depleted BMP4 in 344SQ cells (Fig. 3a) and H157 human lung cancer cells (Additional file 1: Figure S5A) by stable transfection of shRNAs. *Bmp4* knockdown (KD #2 and #3) suppressed expression of its downstream factors, SMAD1 and SMAD5 (Fig. 3b), which may be mediated by the BMP-SMAD autoregulatory loop [15]. *Bmp4*-depleted cells showed retarded growth rates both on two-dimensional (2-D) plastic (Fig. 3c and Additional file 1: Figure S5B) and in Matrigel 3-D cultures (Fig. 3d) relative to control cells (nontargeting control, NTC). In addition, *Bmp4*-KD suppressed cancer cell migration (Fig. 3e and Additional file 1: Figure S5C) and invasion (Fig. 3f) in Boyden chamber assays. Finally, *Bmp4*-KD cells formed smaller primary tumors and metastasized less to the lungs when injected subcutaneously into syngeneic mice (Fig. 3g). These data support the hypothesis that BMP4 promotes cancer cell migration/invasion, tumorigenesis, and metastasis in lung cancer.

### BMP4 is required for cancer cells to form normal acini in Matrigel 3-D culture

**Fig. 4:**
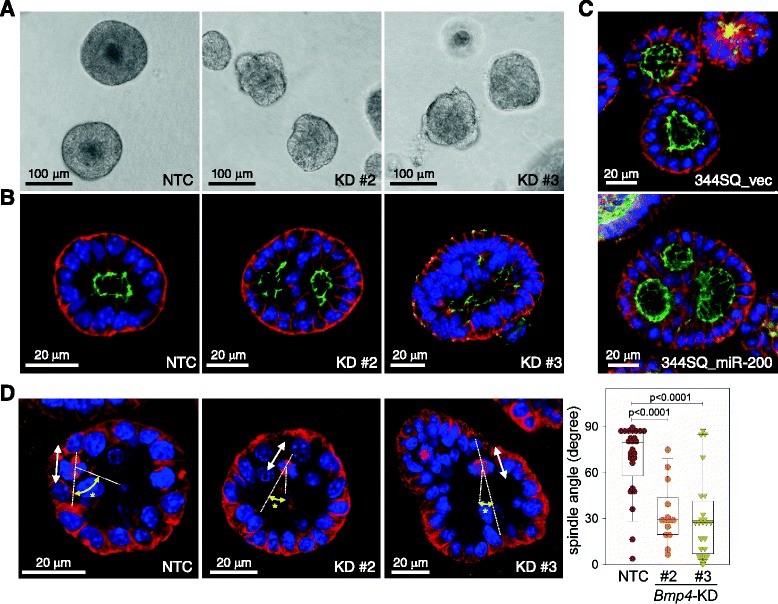
BMP4 is required for normal acinus formation in Matrigel 3-D culture. **a**, **b** Matrigel 3-D culture of 344SQ-NTC and *Bmp4*-KD cells (#2 and #3). Phase contrast (**a**) and confocal microscope (**b**) images of 3-D acini were taken 12 days after cell seeding. Blue: DAPI, red: β-catenin, green: ZO-1. **c** Matrigel 3-D culture of 344SQ_vec and miR-200 cells. Confocal images of 3-D acini were taken 12 days after cell seeding. Blue: DAPI, red: β-catenin, green: ZO-1. **d** Matrigel 3-D culture of 344SQ-NTC and *Bmp4*-KD cells (#2 and #3). Confocal images of 3-D acini were taken 7 days after cell seeding, and spindle angles of dividing cells were measured using ImageJ (http://imagej.nih.gov/ij/). Blue: DAPI, red: α-tubulin. White arrow: direction of cell division; yellow arrow with asterisk: spindle angle. Box-and-whisker plot denotes median and upper/lower quartiles + 1.5 × IQR. p, two-tailed Student’s *t*-test

344SQ cells form well-polarized acini in Matrigel 3-D culture [16]. To examine the effect of BMP4 on acinus formation, we seeded *Bmp4*-KD cells onto the Matrigel layer. After 12 days, 344SQ-NTC cells formed acini with a hollow core; however, *Bmp4*-KD cells formed atypical structures without an evident central hollow (Fig. 4a). Confocal microscopy after staining against basal (β-catenin, red) and apical (ZO-1, green) markers revealed that *Bmp4*-KD colonies had multiple lumens inside the structures (Fig. 4b), which may cause abnormal acinus formation. Similarly, 344SQ_miR-200 cells formed irregular acini with multiple lumens (Fig. 4c), suggesting that the miR-200/BMP4 pathway regulates normal acinus formation in Matrigel 3-D culture. Several reports have demonstrated that misoriented mitotic spindles can cause the formation of multiple-lumen structures in 3-D culture [17, 18]. To check the orientation of mitotic spindles, we stained 3-D acini with an antibody against α-tubulin, which is a component of microtubules in the mitotic spindle. We then measured the spindle angles (the angles between the spindle axis and the line connecting each center of the lumen and the spindle) of cells at metaphase (Fig. 4d). The spindle angles of 344SQ-NTC cells were close to 90°; however, those of *Bmp4*-KD cells varied from 0° to 90°, which is indicative of spindle misorientation. On the basis of these data, we suggest that BMP4 is required for maintaining correct spindle orientation and forming normal lumen structures of 3-D acini in Matrigel culture.

### Non-muscle myosin is a downstream target of BMP4

**Fig. 5:**
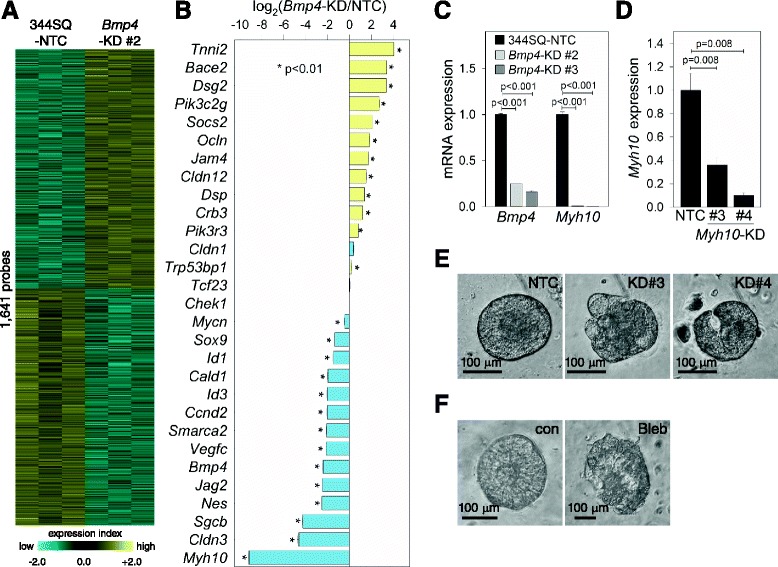
Non-muscle myosin is a downstream target of BMP4. **a** Heatmap of differentially expressed genes (fold change ≥1.5, *p* value <0.05) identified by mRNA microarray of 344SQ-NTC and *Bmp4*-KD (#2) cells. Yellow: increased expression; blue: decreased expression. **b** qRT-PCR of 29 selected genes to confirm the microarray data. Mean + SD, n = 3; p, two-tailed Student’s *t*-test. **c** qRT-PCR of *Bmp4* and *Myh10* in 344SQ-NTC and *Bmp4*-KD cells (#2 and #3). The expression levels were normalized to those of 344SQ-NTC (=1.0). Mean + SD, n = 3; p, two-tailed Student’s *t*-test. **d** qRT-PCR of *Myh10* in 344SQ-NTC and *Myh10*-KD cells (#3 and #4). The expression levels were normalized to those of 344SQ-NTC (=1.0). Mean + SD, n = 3; p, two-tailed Student’s *t*-test. **e**, **f** Matrigel 3-D culture of 344SQ-NTC and *Myh10*-KD cells (KD#3 and #4; E), and 344SQ cells in the presence or absence of blebbistatin (Bleb, 25 μM; F). Phase contrast microscope images of 3-D acini were taken 12 days after cell seeding

To gain insight into genes downstream of BMP4 that mediate its biological functions, we performed global gene expression analysis using a GeneChip® Mouse Genome 430 2.0 Array (Affymetrix). A total of 1641 probes were expressed differentially between 344SQ-NTC and *Bmp4*-KD cells; 810 were up-regulated and 831 were down-regulated in *Bmp4*-KD cells (fold change ≥1.5, *p*-value <0.05; Fig. 5a) (Additional file 2: Table S1). We selected 29 genes and analyzed their mRNA expression levels by quantitative reverse transcription-PCR (qRT-PCR) to confirm the array results (Fig. 5b). The most down-regulated gene among them in *Bmp4*-KD cells was *Myh10*, which encodes non-muscle myosin heavy chain IIB (Fig. 5b and 5c). MYH10 localizes to the contractile ring and regulates cytokinesis during mitosis [19]; therefore, we decided to determine whether non-muscle myosin would influence 3-D acinus formation. Both *Myh10* knockdown (Fig. 5d) and blebbistatin, a selective non-muscle myosin II ATPase inhibitor [19], blocked normal acinus formation in Matrigel 3-D culture (Fig. 5e and 5f), which are the same phenotypes observed in *Bmp4*-KD cells. These data suggest that non-muscle myosin is one of the downstream targets of BMP4 that control normal 3-D morphogenesis.

### JAG2, miR-200, and BMP4 form a regulatory loop

**Fig. 6:**
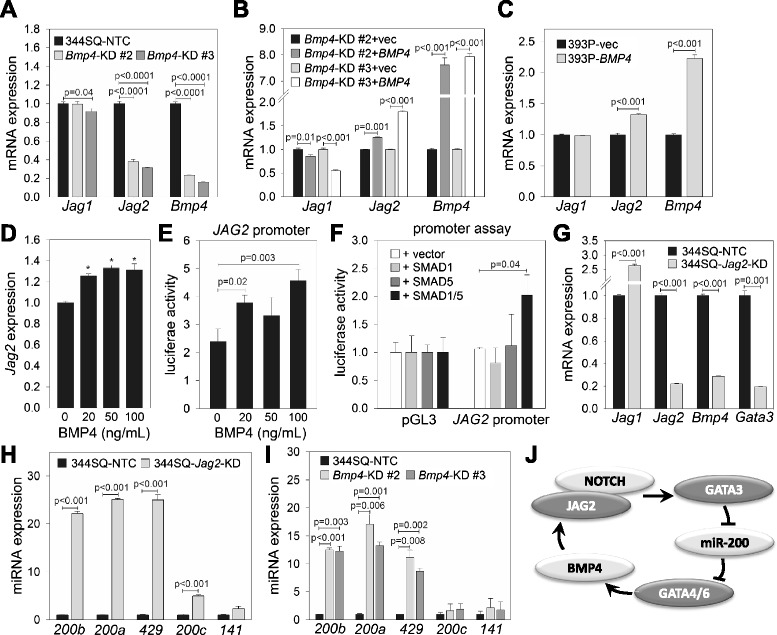
JAG2, miR-200, and BMP4 form a regulatory loop. **a** qRT-PCR of *Jag1*, *Jag2*, and *Bmp4* in 344SQ-NTC and *Bmp4*-KD cells (#2 and #3). The expression levels were normalized to those of 344SQ-NTC (=1.0). Mean + SD, n = 3; p, two-tailed Student’s *t*-test. **b** qRT-PCR of *Jag1*, *Jag2*, and *Bmp4* in 344SQ-*Bmp4*-KD cells transfected with *BMP4* expression vector and control (vec). The expression levels were normalized to those of vector-transfected groups (=1.0). Mean + SD, n = 3; p, two-tailed Student’s *t*-test. **c** qRT-PCR of *Jag1*, *Jag2*, and *Bmp4* in 393P cells transfected with *BMP4* expression vector and control (vec). The expression levels were normalized to those of 393P-vec (=1.0). Mean + SD, n = 3; p, two-tailed Student’s *t*-test. **d** *Jag2* qRT-PCR in 393P cells treated with BMP4 protein. The expression levels were normalized to that of non-treated control (=1.0). Mean + SD, n = 3; *p < 0.01, two-tailed Student’s *t*-test. **e**, **f** Luciferase assay of *JAG2* promoter activity in 393P cells treated with BMP4 protein (E) or co-transfected with SMAD1 and/or SMAD5 expression vectors (**f**). Results were normalized by using a Renilla luciferase vector (pRL-TK). Mean + SD, n = 3; p, two-tailed Student’s *t*-test. **g** qRT-PCR of *Jag1*, *Jag2*, *Bmp4*, and *Gata3* in 344SQ cells transfected with a *Jag2* shRNA (KD) or nontargeting control (NTC) vector. The expression levels were normalized to those of 344SQ-NTC (=1.0). Mean + SD, n = 3; p, two-tailed Student’s *t*-test. **h** qRT-PCR of miR-200 family members (200a, 200b, 200c, 141, and 429) in 344SQ-*Jag2*-KD and 344SQ-NTC. The expression levels were normalized to those of 344SQ-NTC (=1.0). Mean + SD, n = 3; p, two-tailed Student’s *t*-test. **i** qRT-PCR of miR-200 family members in 344SQ-NTC and *Bmp4*-KD (#2 and #3) cells. The expression levels were normalized to those of 344SQ-NTC (=1.0). Mean + SD, n = 3; p, two-tailed Student’s *t*-test. **j** Diagram of a regulatory loop involving BMP4, JAG2, and miR-200

We previously reported that JAG2, a Notch ligand, promotes lung adenocarcinoma metastasis by suppressing miR-200, which is mediated by GATA3 [12]. BMP4 regulates Notch signaling [20]; therefore, we examined the effect of BMP4 on the expression levels of the Notch ligands, *Jag1* and *Jag2*. In *Bmp4*-KD cells, only *Jag2* was decreased by 60 %-70 % (Fig. 6a), which was partially recovered by BMP4 overexpression (Fig. 6b). When overexpressed in 393P cells, BMP4 enhanced *Jag2* mRNA expression (Fig. 6c). Exogenous BMP4 protein also increased *Jag2* mRNA expression (Fig. 6d) and promoter activity (Fig. 6e). Furthermore, SMAD1 and SMAD5, downstream transcription factors of BMP4, enhanced the transcriptional activity of the *JAG2* promoter (Fig. 6f), suggesting that BMP4 is an upstream regulator of *Jag2* transcription. Of interest, *Jag2*-KD suppressed *Bmp4* mRNA expression (Fig. 6g), which may be mediated by enhancing the expression of miR-200 family members, especially, miR-200b/200a/429 (Fig. 6h). In addition, *Bmp4*-KD increased miR-200b/200a/429 expression (Fig. 6i) by removing the negative regulator of miR-200, JAG2. Collectively, we propose a regulatory loop comprising JAG2, GATA3, miR-200, GATA4/6, and BMP4 (Fig. 6j).

## Discussion

In this study, we investigated the function of BMP4, a TGF-β superfamily member, which is down-regulated by miR-200 in murine lung adenocarcinoma cell lines. BMP4 was originally identified as a morphogen involved in early embryonic development, especially in dorsal-ventral axis differentiation [21] and bone and cartilage formation [22]. BMP4 has been reported to exert inhibitory or stimulatory functions during cancer initiation and metastasis, depending on the tissue and cancer type. For example, BMP4 induces differentiation of cancer stem cells, blocks progression of hepatocellular carcinoma [23], and suppresses tumorigenesis of gastric carcinoma [24]. In contrast, BMP4 promotes prostate tumor growth [25] and induces EMT in pancreatic [11] and ovarian cancer cells [26]. Here, we also described the stimulatory effects of BMP4 on lung tumorigenesis and metastasis. The discrepancies in BMP4’s roles in cancer progression may result from a lack of appropriate signaling networks or tissue-specific operation of transcriptional machineries, such as the GATA family of transcription factors, which control BMP4 expression, as confirmed here. The expression of GATA4 varies greatly according to tissue origin, developmental timing, and cancer stage [27], which results in part from context-specific DNA methylation of the GATA4 promoter region [28]. In addition, GATA4 and GATA6 may transactivate different sets of downstream genes depending on the availability of co-activators and co-repressors such as FOG2, NKX2.5, and p300 [27]. Therefore, the tissue specificity of major regulators and effectors of BMP4 may contribute to its cancer type–specific functions.

Recently, we published our findings that the miR-200/ZEB1 negative-feedback loop regulates CD8^+^ tumor-infiltrating lymphocytes by directly targeting PD-L1 expression [29], which strongly supports the idea that modulation of the immune system is a prerequisite to cancer metastasis. It is also noteworthy that miR-200 can regulate a variety of immune-related genes, including cytokines (e.g., *Il11, Ifna1, Csf1, Tgfb2, Il7*), chemokines (e.g., *Cxcl12, Ccl6, Cxcl7*), and their receptors (e.g., *Tlr12, Lepr, Tlr1*) (Fig. 1a). The relevant regulatory mechanisms may be indirect because some of them are up-regulated on miR-200 overexpression, and even down-regulated genes lack miR-200 binding sites on their 3′-UTRs, with the exception of *Tgfb2* [30]. Indirect regulation by miR-200 could be achieved through targeting of transcription factors such as ZEB1 [8], ETS1 [31], and GATA4/6, as proposed herein, which would vastly expand miR-200’s regulatory network.

Up-regulation of BMP4 in metastatic-prone, mesenchymal lung cancer cells was first observed in our previous study, which aimed to identify the target genes of miR-200 systematically through a proteomic analysis coupled with stable isotope labeling by amino acids in cell culture (SILAC) and mass spectrometry [31]. Through this method, we found that BMP4 is among the up-regulated proteins present in conditioned media obtained from 344SQ cells; however, we could not determine how BMP4 is enriched in mesenchymal cancer cells, nor whether BMP4 influences tumorigenesis and metastasis. We have clearly demonstrated here that BMP4 is indirectly suppressed by miR-200 and that BMP4 promotes tumor cell migration/invasion and metastasis. In addition, we integrated BMP4 into the JAG/Notch signaling pathway and proposed a regulatory loop consisting of JAG2, GATA3, miR-200, and BMP4 (Fig. 6h), suggesting that BMP4 interconnects with various cell signaling partners to induce tumorigenesis and metastasis in lung adenocarcinomas. The miR-200b/200a/429 cluster seems to be more influenced by BMP4–JAG2–GATA3 pathway than the miR-200c/141 cluster, which might be caused by differential binding preference of transcription factors and co-regulators.

When cultured on Matrigel, metastatic 344SQ cells grow into an acinus with a central hollow maintaining apical-basal polarity, which responds to pro-metastatic signals and becomes invasive through the matrix [6, 16]. However, non-metastatic murine lung cancer cells (e.g., 344SQ_miR-200 cells) neither form a well-polarized acinus nor change morphology in response to external signals [6], suggesting that the abilities to form acini, maintain polarity, and respond to pro-invasive signals are among the characteristics of metastatic-prone lung cancer cells. Similar to ectopic expression of miR-200, depletion of BMP4 also induced abnormal shape and multiple-lumen formation of 3-D acini (Fig. 4), which could be caused by misorientation of mitotic spindles [17, 18]. CDC42 GTPase and its downstream partners, PARD6B and atypical PKC, coordinate spindle orientation and regulate apical-basal positioning during epithelial morphogenesis in Matrigel 3-D culture [17, 32]; however, CDC42 activity was not affected by *Bmp4* knockdown in 344SQ cells (data not shown). Unexpectedly, one possible clue was obtained from mRNA profiling of *Bmp4* knockdown cells: *Myh10* was significantly down-regulated by depletion of BMP4 (Fig. 5a), which localizes to the contractile ring and controls cytokinesis during mitosis [19]. The central spindle cooperates with the contractile ring during cell division, and defective cytokinesis is closely related to improper spindle orientation [33]; therefore, *Myh10* down-regulation might disrupt polarity and induce multiple-lumen formation in 3-D acini, which is supported by the results that *Myh10* knockdown and a potent myosin II inhibitor, blebbistatin, caused abnormal acinus formation without obvious central hollows (Fig. 5e and 5 f). Moreover, a recent finding suggests that direct targeting of *MYH10* itself by miR-200a inhibits cell migration and tumor growth in meningiomas [34]. More intensive efforts will be required to elucidate the relationship between BMP4 and MYH10 and to clarify the roles of MYH10 in cancer progression.

## Conclusions

In summary, BMP4 functions as a pro-tumorigenic factor in a murine lung cancer model and is regulated by miR-200 and GATA4/6. BMP4 also interacts with the JAG2/NOTCH signaling pathway that controls lung tumorigenesis and metastasis. In addition, BMP4 is essential for normal acinus formation in Matrigel 3-D culture, which is mediated by non-muscle myosin. On the basis of these findings, we propose that BMP4 and BMP receptor antagonists such as DMH2 [35] can be applied to establish prognostic markers or identify therapeutic targets in patients with lung adenocarcinoma.

## Methods

### Cell culture

Murine (307P, 344LN, 344P, 344SQ, 393LN, 393P, 412P, 531LN1, 531LN2, 531LN3, 531P1, 531P2, and 713P) and human (H157) lung cancer cells were cultured in RPMI 1640 (Corning) with 10 % FBS (Sigma-Aldrich). The generation of transfected cells (344SQ_miR-200 and 393P_ZEB1) has been reported previously [6, 16]. Murine *Bmp4* shRNAs (#TG516759) were purchased (Origene) and *Myh10* shRNAs were cloned into the pLKO.1 vector (Addgene) according to the protocol (http://www.addgene.org/tools/protocols/plko/). These shRNAs were introduced into 344SQ cells by viral infection, and after antibiotics selection for more than 2 weeks, stable transfectants were established.

For migration and invasion assays, cells (1x10^5^) were cultured in the upper wells of Transwell® or Matrigel™ chambers (BD Biosciences), respectively, and allowed to migrate toward 10 % FBS in the bottom wells in the presence of mitomycin C (1 μg/ml) to block cellular proliferation. After 16 h of incubation, migrating or invading cells were stained with 0.1 % crystal violet, photographed, and counted. Cellular growth was measured by counting cells using the Countess™ automated cell counter (Life Technologies). Cells were seeded on three-dimensional Matrigel™ (BD Biosciences), and immunofluorescent labeling was performed as described [6]. A Zeiss LSM 510 confocal microscope was used to capture fluorescent images of Matrigel™ cultures. Antibodies against β-catenin (BD Biosciences), ZO-1 (Invitrogen), and α-tubulin (Sigma-Aldrich) were purchased.

### Quantitative reverse transcription-PCR (qRT-PCR)

Total RNA was isolated from cells by using TRIzol® (Life Technologies) according to the manufacturer’s protocol. After reverse-transcription with qScript™ cDNA SuperMix (Quanta Biosciences), quantitative PCR assays were performed to analyze mRNA levels by using a SYBR-Green-based system (Applied Biosystems). mRNA levels were normalized to levels of *Rpl32*. Primer sequences are listed in Additional file 2: Table S2. MicroRNA levels were quantified by using TaqMan™ miRNA Assays (Applied Biosystems) according to the manufacturer’s protocol and normalized to levels of snoRNA-135.

### Western blotting

Cells were lysed in 50 mM Tris-HCl (pH 7.4), 150 mM NaCl, 1 mM EDTA, and 1 % Triton X-100 with protease/phosphatase inhibitors (Sigma-Aldrich). Cell lysates were separated by SDS-PAGE, transferred onto a PVDF membrane, and then incubated with primary antibodies and horseradish peroxidase (HRP)-conjugated secondary antibodies (Cell Signaling Technology). Protein bands were visualized with Pierce ECL Western blotting substrate (Thermo Scientific). Antibodies against BMP4 (Santa Cruz Biotechnology), β-actin (Sigma-Aldrich), pSMAD1/5 and SMAD1/5 (Cell Signaling Technology) were purchased.

### RNA polymerase II-ChIP assay

Cells were cross-linked with 1 % formaldehyde and incubated in lysis buffer (50 mM Tris-HCl [pH 8.1], 1 % SDS, 10 mM EDTA, and protease inhibitor cocktail) on ice for 10 min and sonicated (Cole-Parmer GEX-130 sonicator; pulse mode for 10 s followed by no pulsing for 10 s, for 20 cycles, at 50 % power). Chromosomal DNA from 344SQ_vector or 344SQ_miR-200 cells was immunoprecipitated with anti-RNA polymerase II antibody (Millipore) or control mouse IgG. DNA was eluted and purified with a PCR purification kit (Qiagen), and a quantitative PCR analysis was carried out to amplify the *Bmp4* promoter region using specific primers (forward: 5′-CTGCTCACAGCCTGTTTCAA-3′, reverse: 5′-TGGGCTTCCCTGAGTTTAGA-3′).

### Luciferase reporter assay

For the promoter assays, the murine *Bmp4* promoter (-1884 to +266 bp from the transcription start site) was isolated by PCR from TC-1 murine ES cell genomic DNA and ligated into the pGL3-basic vector (Promega). 393P cells were seeded on 24-well plates (1x10^5^ cells/well) one day before transfection, then co-transfected with the promoter reporter (500 ng) and *GATA4* or *GATA6* plasmid (gifts from Dr. David Berger, the Michael E. DeBakey VA Medical Center). The human *JAG2* promoter construct (-573 to +476 bp) was purchased (SwitchGear Genomics), subcloned into pGL3-basic, and then co-transfected with *SMAD1* (Addgene #14044) and *SMAD5* (Addgene #11744) expression plasmids. The pRL-TK vector (50 ng, Promega) was co-transfected as an internal control. Recombinant human BMP4 protein (R&D Systems) was added to the cultures to activate BMP4 signaling. For the 3′-UTR assays, murine *Gata4* or *Gata6* 3′-UTR was amplified by PCR from genomic DNA and ligated into the pCI-neo-hRL vector (a gift from Dr. Gregory Goodall, University of Adelaide, Australia). Human *ZEB1* 3′-UTR (a gift from Dr. Gregory Goodall) was used as a positive control. 3′-UTR reporters (500 ng) and pGL3-control (50 ng, Promega) were co-transfected into 344SQ cells seeded on 24-well plates (1x10^5^ cells/well) in the presence or absence of Pre-miR™ miR-200 precursors (5 nM, Ambion). A PCR-based site-directed mutagenesis strategy was carried out to generate mutant constructs. At 48 h after transfection, luciferase activity was measured with use of the Dual-Luciferase Reporter Assay System (Promega).

### Mouse experiments

Before initiation, all proposed mouse studies were submitted to and approved by the Institutional Animal Care and Use Committee (IACUC) of The University of Texas MD Anderson Cancer Center. Mice were cared for and were euthanized according to the standards set forth by the IACUC. Syngeneic (129S2/SvPasCrl) mice (n = 8 to 10 per group) were injected subcutaneously in the right flank with 344SQ cells (1x10^6^ cells per mouse) that had been stably transfected with *Bmp4* shRNAs (#2 and #3) or with NTC cells. Mice were monitored daily for tumor growth, euthanized at 6 weeks after injection or at the first sign of morbidity, and necropsied to isolate primary tumors and to determine the sites of metastases.

### mRNA expression profiling

Snap-frozen cells (344SQ-NTC and 344SQ-*Bmp4* shRNA #2) were shipped to Asuragen for processing and analysis with use of GeneChip® Mouse Genome 430A 2.0 Array chips (Affymetrix). Data processing and determination of differentially expressed genes were carried out essentially as described [36].

### Statistical analysis

Data were analyzed with Student’s *t*-test and Spearman’s rank correlation test by using GraphPad Prism.

## Additional files

Additional file 1: Figure S1. Negative correlation between *Bmp4* and miR-200 family members in murine lung adenocarcinoma cells. **Figure S2.** Negative correlation between *BMP4* and miR-200 family members in human lung adenocarcinomas. **Figure S3.** Negative correlation between *BMP4* and miR-200 family members in human breast invasive carcinomas. **Figure S4.** *Bmp4* is down-regulated in miR-200-overexpressing cells. **Figure S5.** *BMP4* knockdown suppresses cancer cell growth and migration. (PDF 933 kb) Additional file 2: Table S1. Differentially expressed genes between 344SQ-NTC and 344SQ-*Bmp4*-KD cells. **Table S2.** qRT-PCR primers used in this study. (ZIP 205 kb)

## Abbreviations

BMP4: Bone morphogenetic protein 4
miR: microRNA
EMT: Epithelial-mesenchymal transition
UTR: Untranslated region
qRT-PCR: Quantitative reverse-transcription polymerase chain reaction
KD: Knockdown
NTC: Nontargeting control.
