# Supplementary material for: BMP4 depletion by miR-200 inhibits tumorigenesis and metastasis of lung adenocarcinoma cells
Source: Mol Cancer. 2015 Sep 22;14:173. doi: 10.1186/s12943-015-0441-y (PMC4580148; doi:10.1186/s12943-015-0441-y)
Supplement: Additional file 1: Figure S1. — Negative correlation between Bmp4 and miR-200 family members in murine lung adenocarcinoma cells. Figure S2. Negative correlation between BMP4 and miR-200 family members in human lung adenocarcinomas. Figure S3. Negative correlation between BMP4 and miR-200 family members in human breast invasive carcinomas. Figure S4. Bmp4 is down-regulated in miR-200-overexpressing cells. Figure S5. BMP4 knockdown suppresses cancer cell growth and migration. (PDF 933 kb) [file 12943_2015_441_MOESM1_ESM.pdf]

Supplementary Figure legends

Figure S1. Negative correlation between BMP4 and miR-200 family members in murine lung adenocarcinoma cells.

Quantitative RT-PCR (qRT-PCR) of *Bmp4* and miR-200 family members (miR-200a, 200b, 141, and 429) in 13 murine lung adenocarcinoma cells. r and p, one-tailed Spearman's rank correlation test.

Figure S2. Negative correlation between BMP4 and miR-200 family members in human lung adenocarcinomas.

mRNA and miRNA microarray data of human lung adenocarcinomas (n=32) were obtained from NCBI Gene Expression Omnibus database (GSE63459 and GSE63805, respectively). r and p, one-tailed Spearman's rank correlation test.

Figure S3. Negative correlation between BMP4 and miR-200 family members in human breast invasive carcinomas.

mRNA and miRNA expression data of human breast invasive carcinomas (TGCA, Nature 2012, n=285) were obtained from cBioPortal (http://www.cbioportal.org/). r and p, one-tailed Spearman's rank correlation test.

Figure S4. BMP4 is down-regulated in miR-200-overexpressing cells.

(A) qRT-PCR of miR-200 family members (200a, 200b, and 200c) in 344SQ transfected with miR-200b/200a/429. The expression levels were normalized to those of 344SQ_vec (=1.0). Mean+SD, n=3; p, two-tailed Student's *t*-test. (B) qRT-PCR of *Bmp4* and *Zeb1* in 531LN2

transfected with miR-200b/200a/429 (miR-200). The expression levels were normalized to those of 531LN2_vec (=1.0). Mean+SD, n=3; p, two-tailed Student's *t*-test.

Figure S5. BMP4 knockdown suppresses cancer cell growth and migration. (**A**) qRT-PCR of *BMP4* in H157 transfected with *BMP4* shRNAs (KD #3 and #5) or nontargeting control (NTC) vector. The expression levels were normalized to that of NTC (=1.0). Mean+SD, n=3; p, two-tailed Student's *t*-test. (**B**) MTT assay of H157-*BMP4*-KD and NTC cells over 4 days. Mean±SD, n=4; p, two-tailed Student's *t*-test. (**C**) Soft agar assays of H157-*BMP4*-KD and NTC cells over 2 weeks. Colonies (> 200 μm in diameter) of three randomly selected fields were counted using ImageJ. Mean+SD, n=3; p, two-tailed Student's *t*-test. (**D**) Migrated cells in Boyden chambers were photographed and counted. Mitomycin C (1 μg/mL) was added to the culture media to block cell proliferation. Mean+SD, n=3; p, two-tailed Student's *t*-test.

Murine lung adenocarcinoma cells (n=13)

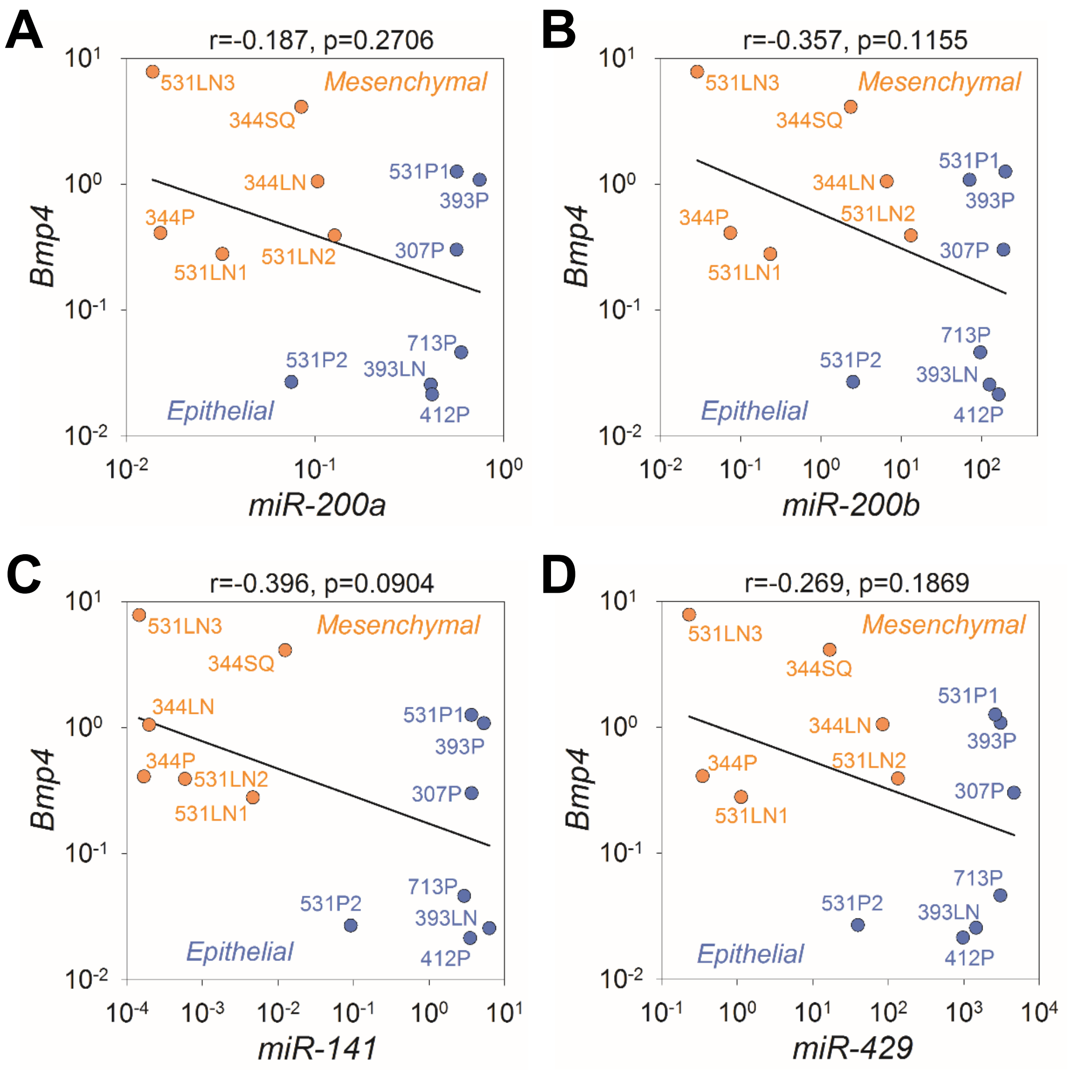

Figure S1

Human lung adenocarcinomas (n=32)

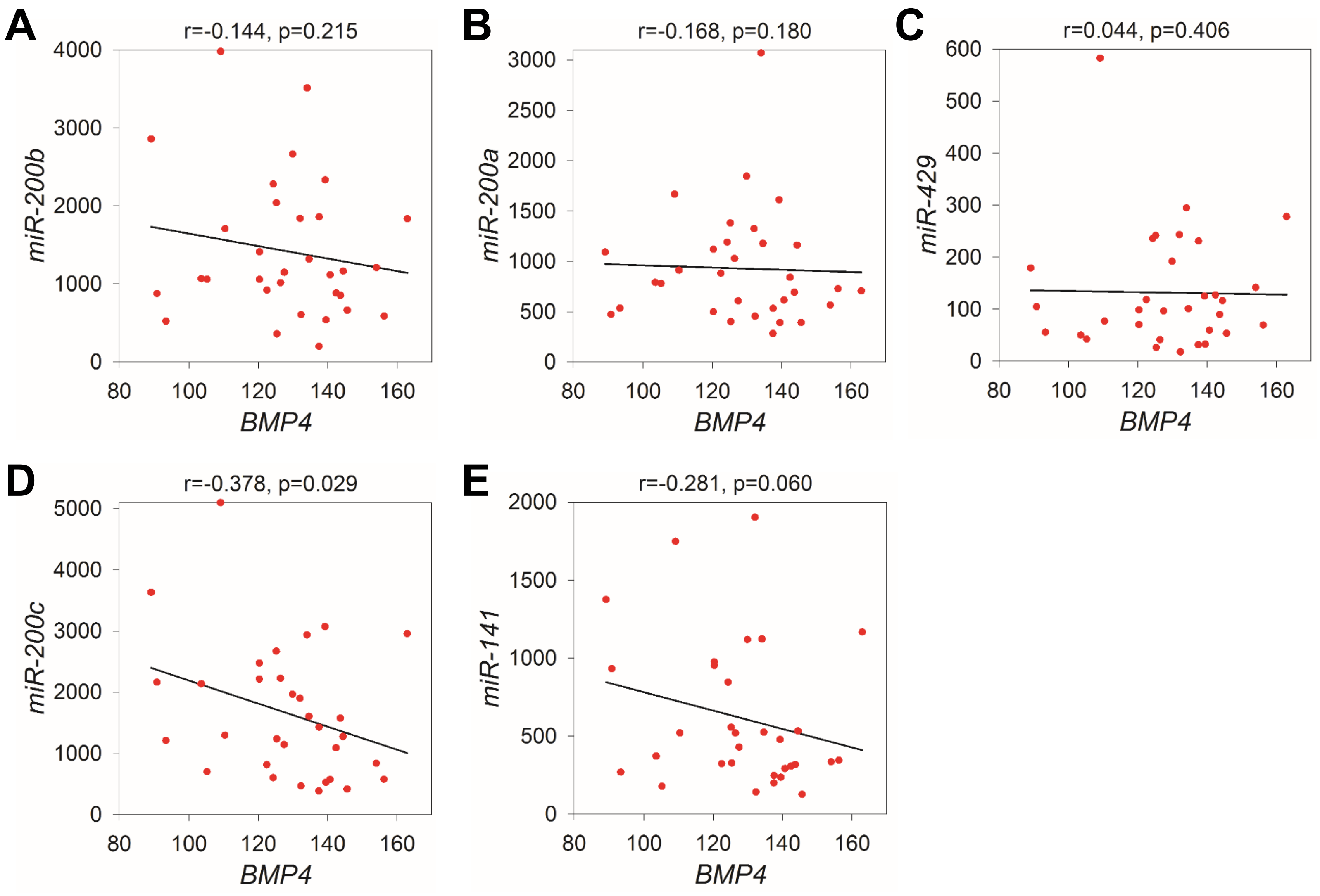

Figure S2

Human breast invasive carcinomas (n=285)

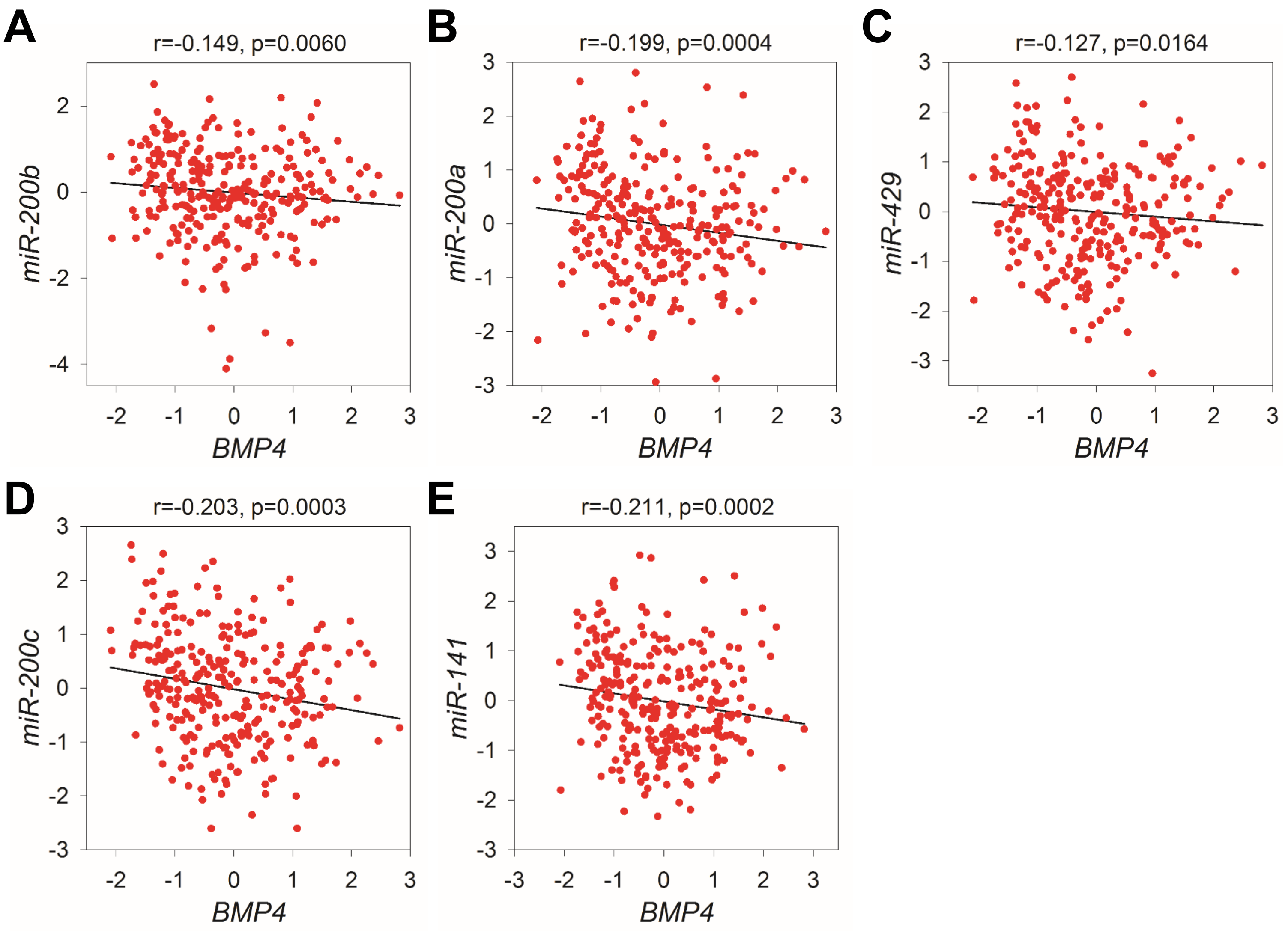

Figure S3

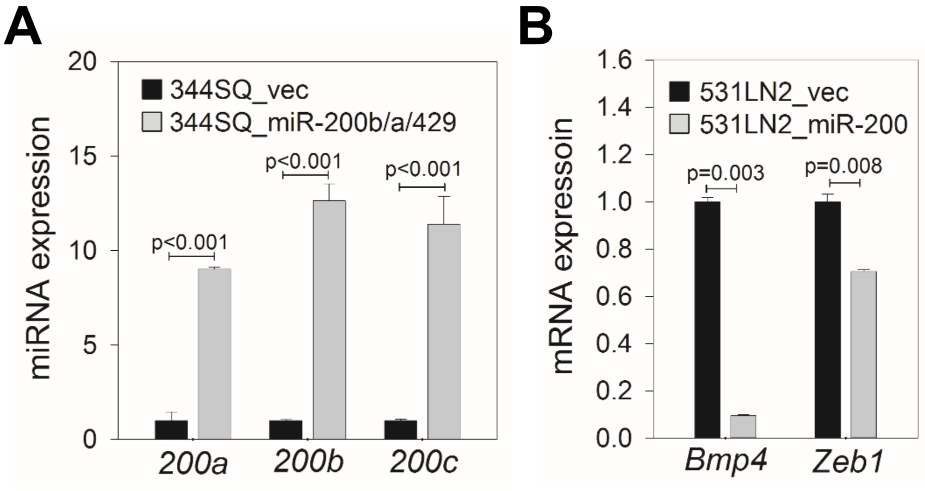
A
miRNA expression
20
15
10
5
0
344SQ_vec
344SQ_miR-200b/a/429
p<0.001
p<0.001
p<0.001
200a
200b
200c
B
mRNA expressoin
1.6
1.4
1.2
1.0
0.8
0.6
0.4
0.2
0.0
531LN2_vec
531LN2_miR-200
p=0.003
p=0.008
Bmp4
Zeb1

Figure S4

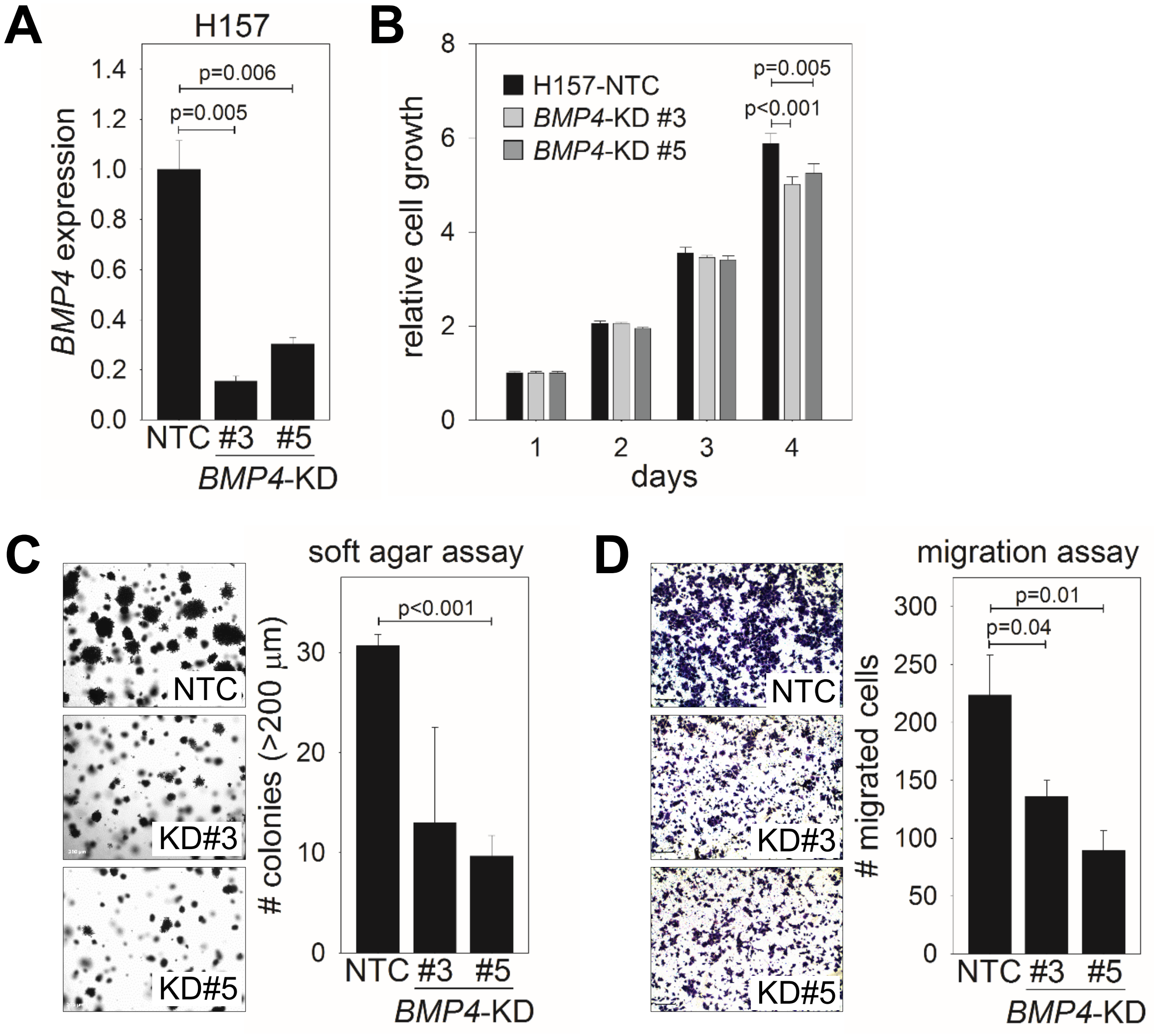
A
H157
BMP4 expression
p=0.006
p=0.005
1.4
1.2
1.0
0.8
0.6
0.4
0.2
0.0
NTC #3 #5
BMP4-KD
B
relative cell growth
H157-NTC
BMP4-KD #3
BMP4-KD #5
p=0.005
p<0.001
8
6
4
2
0
1 2 3 4
days
C
NTC
KD#3
KD#5
soft agar assay
colonies (>200 μm)
p<0.001
30
20
10
0
NTC #3 #5
BMP4-KD
D
NTC
KD#3
KD#5
migration assay
# migrated cells
p=0.01
p=0.04
300
250
200
150
100
50
0
NTC #3 #5
BMP4-KD

Figure S5
