# Supplementary material for: BMP4 depletion by miR-200 inhibits tumorigenesis and metastasis of lung adenocarcinoma cells
Source: Mol Cancer. 2015 Sep 22;14:173. doi: 10.1186/s12943-015-0441-y (PMC4580148; doi:10.1186/s12943-015-0441-y)
Supplement: Additional file 2: Table S1. — Differentially expressed genes between 344SQ-NTC and 344SQ-Bmp4-KD cells. Table S2. qRT-PCR primers used in this study. (ZIP 205 kb) [file 12943_2015_441_MOESM2_ESM.zip › Additional file 2/Table S2. primers.pdf]

Table S2. qPCR primers used in this study.

gene	*forward (5′→3′)*	*reverse (5′→3′)*
Bmp4	ACCCGCAGAAGGGCCAAACG	TGGACCGCTGTGGGTGATGC
Bmpr2	CTTAAGCGGTGGCGCCCGT	TCCAGCGGCTCGGTGGAGAT
Alk2	TGTGTCGGGAAGGGCCGGTA	CTTGCCAGCTGCCCCTCCAT
Alk3	CAGGCAGTGAGCGCTCTAGTCA	TGCAGTGATGGCAGCGTGGG
Alk6	TTTGGGCCAGCCCTGCTCTG	TGCGTGCGCATTGACTCTGC
Gata4	CTCTTCAAATTCCTGCTCGG	CAGCCTTTTACTTTGCTGGC
Gata6	CTACACAAGCGACCACCTCA	TCTCCCACTGCAGACATCAC
Zeb1	GCTCAGCCAGGAACCCGCAG	TGGGCACCCTCTGCCACACA
Zeb2	AGGCGCGAGAGAAAGGGCAC	CCCGGTTCATCAGCAGCTCGG
Snai1	CCCAAGGCCGTAGAGCTGA	GCTTTTGCCACTGTCCTCATC
Snai2	ATCCTCACCTCGGGAGCATA	TGCCGACGATGTCCATACAG
Cdh1	TACGGCGGTGGTGAGGACGA	GCCACACGGGGGAGACTTGC
Cdh2	CCTCCAGAGTTTACTGCCATGAC	CCACCACTGATTCTGTATGCCG
Cldn3	CACCTGACTACCGGGCCTAG	GGTTTCTTTGTCCATTCGGCT
Inadl	GGAAGATTTGCCTCTGTACCGAC	GCTGAAGTTCGGTGTCTCCTCT
Pard6b	TTTCCACCGCCAATCCACTGCT	GCTGATGACGATGTGAGGCTTC
Prkci	CAACTGCAAACTGCTGGTTCA	TCAATTGTGACCAGCTTGTGG
Scrib	CCCAGGAAGGCCGTGGCAAG	GCCTCAGCCAGCCGTTCCAA
Vim	GCGTGCGGCTGCTTCAAGAC	ATGGCGTCGGCCAGCGAGAA
Fn1	GGGCTGGCGCTGTGACAACT	CGGGACTGGGTTCAGCAGCC
Jag1	TACTGTGGGACTCATCAGCCC	AGTGTTGCTACATGTTCCCCG
Jag2	GGATGGGCAAAGAATGCAAA	GCAAATTACATCCTTGTTTACACACG
Gata3	GTTCCTCCGACCCCTTCTAC	TTCATGATACTGCTCCTGCG
Bmp2	AACACCGTGCGCAGCTTCCATC	CGGAAGATCTGGAGTTCTGCAG
Bmp3	TAACACGGTCCGCAGCTTCAGA	TGTGGCTGACAAAATGTTCTCCG
Bmp3b	TACAACCGAAGAGGTGCTCCAC	GGTTCTGAGTAGAAGTGGAAGGC
Bmp5	GGCTTACAGCTCTGTGCAGAGA	GGATCGAAGAAGTACCTCGCTTG
Bmp6	CTTTCCTCAACGACGCGGACAT	CCTCAGGAATCTGGGATAGGTTG
Bmp7	GGAGCGATTTGACAACGAGACC	AGTGGTTGCTGGTGGCTGTGAT
Bmp8a	GTGGTCCAAGAGCACTCCAACA	CAGGTCCTTGTGATGGTTCAGC
Bmp8b	GTGGTCCAAGAGCACTCCAACA	GGTCCTTGTGATGGTTCAGCAG
Bmp9	TCCAACATCGTGCGGAGCTTCA	CAGGAGACATAGAGTCGGAGCT
Bmp10	CACCAGAGTACATGCTGGAGCT	GGATAGACACATTGAAGAGGAGAG
Bmp11	TTTCGCCAGCCACAGAGCAACT	CTCTAGGACTCGAAGCTCCATG
Bmp12	CATGATGTCGCTTTACAGGAGCC	AGAGGCTGGATACGTCGAACAG
Bmp13	CACTAGCTTTGTAGACAGAGGAC	CCTGGCGATAAAGCCTTAGCTC
Bmp14	GGAGGTAACAGCAGCGTGAAGT	CCTTCTCCAAGGCACTGATGTC
Bmp15	GATTGGAGCGAAAATGGTGAGGC	GCTACCTGGTTTGATGCTAGAGG
Tnni2	AACGGGACCTGCGTGACGTG	TTCCGGCCCTCCATGCCAGA
Bace2	CTGATCGGGACCCCTCCGCA	GGCACCTGCCACAGCGAAGT
Dsg2	ACCAGCCTCCTGGAACGGCA	TTCTCCCGCGTTTCCGCTCG
Pik3c2g	AGCTGCTGGACTGCCTCCCA	TTTGCAAGGGGCGACGAGGC
Socs2	AGGTCCACTGGCCCCAGCTC	TCCGCTCCATTCCCGGAGGG
Ocln	GCATGTCCGGCCGATGCTCT	TGGGGGCGACGTCCATTTGT

Jam4	GAGGAAGGCCCAGCACTGCC	GGCCACTGCCAGCAGGATGA
Cldn12	GCGCAGACAGGCTGCTTGGA	GCCCATGGCAGGAGGGCTTG
Dsp	GGTACGTGACGGGCCCAGGA	GGCCCACGGAAGGGACAAGC
Crb3	CGGACCCTTTCACAAATAGCA	CGTTGGACTCATCACCTGGG
Pik3r3	CCGGGATCAGCACCTTGTATGGC	TCAGGCGCCTCTGCCTCACT
Cldn1	TGCAGGCAACCCGAGCCTTG	TGCGATCAGCCCCAGCAGGA
Trp53bp1	TGGGCAGCGTGCAGAAACCTC	GGTCCATCTGCTCCCCTGGCA
Tcf23	AGAGCCACCTCTGCCCCTCG	TCCGTTCCCGTGCTGCGTTC
Chek1	TGGCAGCTGGCAAAGGACTGC	GGCACTGCCATGACTCCAAGCA
Mycn	CTGGGTTGGAGCCGAACGAGC	GGCGGGAGGGGGTTGTCCTC
Sox9	CACACGTCAAGCGACCCATGAA	TCTTCTCGCTCTCGTTCAGCAG
Id1	GCAGGCCCTAGCTGTTCGCT	GTACCACCTCGCCCGCTGTC
Cald1	TTCAGCCCCAGCCGTTCAGG	GCCTCTTGCCGGCTTCCACT
Id3	CTGGCACCTCCCGAACGCAG	AGTCCCAGGGTCCCAAGCGG
Ccnd2	GCAAGGAACCGTGGGTGCCA	ACCTCCCCGGCATGTCCCTT
Smarca2	GCAGAACCGAATGCTTTCTT	AGTGAACTCACTGGGGAGGA
Vegfc	AGCATTGTGATCCAGGACTGT	GGACACAGCGGCATACTTCT
Nes	GTGCAGCGCGACAACCTTGC	TCCTCGATGGTCCGCTCCCG
Sgcb	GGCACGGTGATGGTCAGCCC	TGACTGGTCCCCGCTGGAGG
Myh10	GGGCGTGGAGATGGCCCAATG	CGCCGGGCTCCTTTAGCACTG
Rpl32	GGAGAAGGTTCAAGGGCCAG	TGCTCCCATAACCGATGTTTG
